# Hyperbaric oxygen therapy for nonischemic diabetic ulcers: A systematic review

**DOI:** 10.1111/wrr.12776

**Published:** 2019-11-26

**Authors:** Rutger C. Lalieu, Robin J. Brouwer, Dirk T. Ubbink, Rigo Hoencamp, René Bol Raap, Rob A. van Hulst

**Affiliations:** ^1^ Hyperbaar Geneeskundig Centrum Rijswijk The Netherlands; ^2^ Department of Surgery Alrijne Hospital Leiderdorp The Netherlands; ^3^ Academic Medical Center, Department of Surgery Amsterdam University Medical Centers Amsterdam The Netherlands; ^4^ Ministry of Defense Defense Healthcare Organization Utrecht The Netherlands; ^5^ Department of Surgery Leiden University Medical Center Leiden The Netherlands; ^6^ Academic Medical Center, Department of Anesthesiology Amsterdam University Medical Centers Amsterdam The Netherlands

## Abstract

Diabetic foot ulcers are a common complication of diabetes, which affects 25% of patients and may ultimately lead to amputation of affected limbs. Research suggests hyperbaric oxygen therapy improves healing of these ulcers. However, this has not been reflected in previous reviews, possibly because they did not differentiate between patients with and without peripheral arterial occlusive disease. Therefore, we performed a systematic review of published literature in the MEDLINE, Embase, and Cochrane CENTRAL databases on nonischemic diabetic foot ulcers with outcome measures including complete ulcer healing, amputation rate (major and minor), and mortality. Seven studies were included, of which two were randomized clinical trials. Two studies found no difference in major amputation rate, whereas one large retrospective study found 2% more major amputations in the hyperbaric oxygen group. However, this study did not correct for baseline differences. Two studies showed no significant difference in minor amputation rate. Five studies reporting on complete wound healing showed no significant differences. In conclusion, the current evidence suggests that hyperbaric oxygen therapy does not accelerate wound healing and does not prevent major or minor amputations in patients with a diabetic foot ulcer without peripheral arterial occlusive disease. Based on the available evidence, routine clinical use of this therapy cannot be recommended. However, the available research for this specific subgroup of patients is scarce, and physicians should counsel patients on expected risks and benefits. Additional research, focusing especially on patient selection criteria, is needed to better identify patients that might profit from this therapy modality.

**Abbreviations**
ABI: ankle‐brachial pressure index
ATA: atmosphere absolute
DFU: diabetic foot ulcer
HBOT: hyperbaric oxygen therapy
PAOD: peripheral arterial occlusive disease
TBI: toe‐brachial pressure index
TBP: toe blood pressure
TcpO_2_: transcutaneous oxygen pressure measurement
UHMS: Undersea and Hyperbaric Medical Society

## INTRODUCTION

Diabetes is a growing global health issue. Currently, an estimated 8.5% of the adult population now suffers from this disease.^1^ With it comes a plethora of complications, including increased risk of heart attack, stroke, and premature death.^2^ Diabetic foot ulcers (DFUs) are one of the most common diabetes‐related complications, affecting up to 25% of patients and leading to major personal and economic consequences.^3^, ^4^ For example, the reported mortality rates associated with DFUs rival or exceed those of some common cancers.^5^

The development of DFU is multifactorial, with the most important risk factors being neuropathy and vasculopathy. It is estimated that the majority of ulcers are of neuropathic rather than vascular origin.^6^ However, even in neuropathic ulcers, these changes are most notable as microvascular disease.^7^ Once present, ulcer healing is intrinsically impaired due to the physiological and immunological changes associated with diabetes. Despite optimal treatment, previous studies have shown that 19% to 35% of diabetic ulcers remain unhealed,^8^, ^9^, ^10^ considerably increasing the risk of amputation of the affected limb.^11^ Even if complete wound healing has been achieved, there is still the risk of recurrence: 40% of patients develop a recurring wound within 1 year.^4^, ^12^

When it comes to chronic wounds, local tissue hypoxia is one of the most important sustaining factors.^13^ Hyperbaric oxygen therapy (HBOT) aims to correct this hypoxic state in order to improve wound healing and, consequently, to prevent amputations.

The definition of HBOT is the breathing of 100% oxygen under an atmospheric pressure greater than sea level, or one atmosphere absolute (ATA). The Underwater and Hyperbaric Medical Society (UHMS) states that a pressure of at least 1.4 ATA is required to have a clinical effect.^14^ Generally, the treatment is considered safe^15^ and cost‐effective.^16^, ^17^ In Europe, there are several recognized indications for the therapy, including osteoradionecrosis of the mandible, delayed radiation injury, compromised skin grafts, and diabetic foot lesions.^18^ The therapy is usually applied daily for several weeks and can be performed in monoplace or multiplace chambers (i.e. accommodating a single patient or several). It provides several physiological effects beneficial for wound healing, such as increased angiogenesis and leukocyte activity, improved collagen deposition and reduction of edema.^19^, ^20^

Despite these advantages, the application of HBOT remains controversial. The evidence on clinical effectiveness is low, which is one of the reasons why HBOT is frequently used as a “last‐resort” treatment option in the Netherlands. It is generally considered to be cumbersome and time‐consuming and, additionally, not all physicians have ready access to a hyperbaric chamber. For the same reasons, few randomized clinical trials (RCTs) have been performed on the effectiveness of HBOT for diabetic and/or ischemic ulcers.^21^ Other research is considered to be of questionable quality.^22^ The quality of evidence could be bolstered by the use of a control group, but since there is no consensus on the most valid sham treatment^23^, ^24^ this is not easily implemented.

Previous systematic reviews of the available literature have been inconclusive about the efficacy of HBOT. A Cochrane review by Kranke et al.^21^ concluded that HBOT leads to improved wound healing after 6 weeks, but not after 12 months, and does not reduce major amputation rate. In non‐ischemic DFUs, O'Reilly et al.^25^ found that treatment with HBOT in randomized trials reduced the risk of major amputation but did not significantly affect wound healing. A systematic review by Stoekenbroek et al.^26^ found that none of the relevant RCTs reported significant differences, neither in wound healing nor in amputation rates. All reviews, based on RCTs, concluded that there was not enough evidence to support routine use of HBOT for DFUs in clinical practice.

The inconclusiveness of the previous reviews might, at least in part, be explained by the inclusion of both patients with and without peripheral arterial occlusive disease (PAOD). Often, no distinction was made between these groups when reporting on wound healing or amputation rate, despite the varying pathophysiology of ulcers^6^, ^27^ and the fact that PAOD in itself is a risk factor for major amputation.^28^, ^29^

Hence, the current systematic review aimed to evaluate the current scientific data on the benefits and harms of HBOT adjunctive to local and/or systemic treatments regarding wound healing and amputation rates in patients with DFUs without PAOD.

## METHODS

The review protocol was developed beforehand. The review was conducted and described according to the Preferred Reporting Items for Systematic reviews and Meta‐Analyses (PRISMA) statement.^30^, ^31^

### Search strategy

A clinical librarian helped develop a search strategy to identify the literature concerning diabetic wounds and HBOT using the Embase, MEDLINE, and Cochrane CENTRAL databases, from inception up to September 2018. The search included MeSH and free text terms as 'ulcer,' 'diabetes mellitus,' and 'hyperbaric oxygen therapy' and synonyms. The complete search strategy is supplemented in Appendix A.. Hand search was conducted of references in eligible studies and also of nonpublished trials from digital sources (e.g. http://www.clinicaltrials.gov). No language restriction was applied to prevent publication bias.

### Inclusion and exclusion criteria

Two researchers (RB and RL) independently screened the titles and abstracts of the articles found. Comparative studies were included if they concerned patients with type I or II diabetes who had a DFU without PAOD, and who received HBOT in addition to standard wound care. PAOD was defined as an ankle‐brachial pressure index (ABI) ≤0.9, a toe‐brachial pressure index (TBI) ≤0.70, a toe blood pressure (TBP) <30 mmHg or a transcutaneous oxygen pressure measurement (TcpO_2_) on the dorsum of the foot <30 mmHg.^32^, ^33^ If studies did not provide criteria that measured PAOD, or did not discern ischemic and nonischemic wounds, the corresponding author was emailed to provide these data. If there was no response or the data were not available, the study was excluded. In case of discrepancies, consensus was reached through discussion among authors.

### Data collection

Study characteristics and outcome measures were extracted by two reviewers (RB and RL) independently and entered into predefined electronic tables for the purpose of data checking by the authors. Study characteristics reported were number of patients included, details of HBOT treatment including (mean) number of sessions, outcome measures, type of study design, and year of publication. Primary outcomes of interest were complete wound healing and amputation rate (major (i.e. above the ankle joint) or minor). Secondary outcomes of interest were amputation‐free survival, mortality, any measure of quality of life (QoL) as reported by the authors, number of sessions performed, adverse events, and costs of HBOT. Any discrepancies were resolved by discussion. When consensus was reached, the final data were entered into the Review Manager 5.3 software package (Copenhagen: The Nordic Cochrane Centre TCC, 2014).

### Quality assessment

Two of the authors (RB and RL) independently assessed the quality of the selected studies using the Cochrane checklist^34^ for randomized studies and the ROBINS‐I checklist^35^ for non‐randomized interventional studies. These checklists assess the risk of bias on several domains, including selection bias, allocation bias and performance bias. Risks scores on the ROBINS‐I scale were judged as 'low,' 'moderate,' 'serious' or 'critical,' or if not enough information was available, as 'NI' (no information). The reviewers calculated total scores for each study (columns) and each domain (rows). Again, any differences in assessment were resolved by discussion.

### Statistical analysis

Results are given as means with standard deviations (SD) for normally distributed data or medians with interquartile ranges (IQR) for nonnormally distributed data. Percentages are provided when describing proportions of the study population. Differences between treatment groups are presented as mean differences (MD) or risk differences (RD) with their 95% CI. For significant differences, the number needed to treat (NNT) or number needed to harm (NNH) are also provided.

Data were pooled if clinically homogeneous and if statistical heterogeneity was limited, as expressed by an I^2^ statistic of <75%. A fixed‐effects model was used if I^2^ < 25% and a random effects model if 25% ≤ I^2^ < 75. Both RCTs and observational studies were to be included in the meta‐analyses but are shown separately for a more in‐depth evaluation of the available evidence.^36^ Meta‐analysis, if meaningful, was performed using the Review Manager 5.3 software package (Copenhagen: The Nordic Cochrane Centre TCC, 2014).

## RESULTS

**Figure 1:**
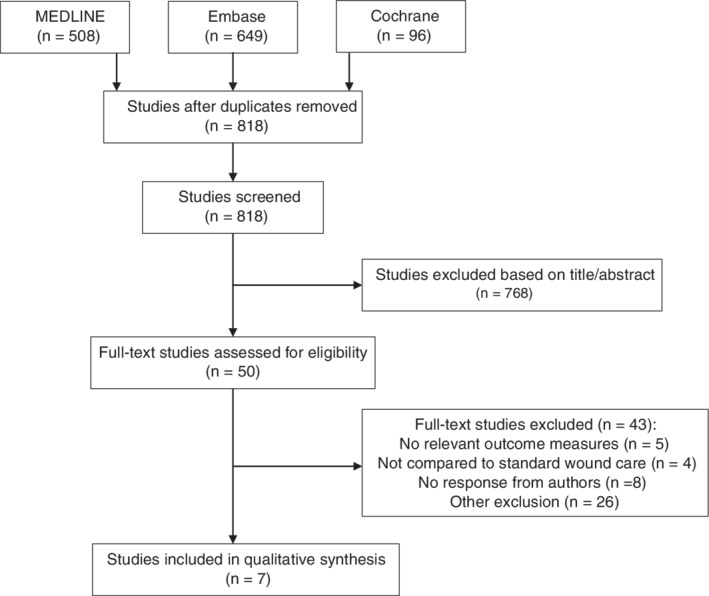
PRISMA flowchart for meta‐analysis up to October 1, 2018.

The literature search yielded a total of 818 eligible articles; see also Figure 1. After screening, six studies^37^, ^38^, ^39^, ^40^, ^41^, ^42^ met the inclusion criteria for PAOD and were selected for qualitative analysis. These studies included two RCTs,^37^, ^40^ two prospective studies,^38^, ^42^ and two retrospective studies.^39^, ^41^ Akgül et al.^43^ provided additional data on patients without PAOD in a retrospective study and was therefore included as well. A total of 6.438 patients were included in this review.

Eight studies,^44^, ^45^, ^46^, ^47^, ^48^, ^49^, ^50^, ^51^ including two RCTs,^46^, ^50^ were excluded because we received no response on our request for additional data from the authors. One of the larger RCTs performed by Fedorko et al.^52^ was excluded as well. In contradiction to the study protocol, the need for amputation was not evaluated by a vascular surgeon in person but by using photographs of the wounds, and evaluations were made by a single, albeit experienced, surgeon. No actual amputations were performed in this study. Therefore, the exclusion of this study appears justified.^53^, ^54^, ^55^

### Characteristics of included studies

Table 1 gives an overview of the characteristics of the included studies. HBOT protocols varied between 60 and 120 minutes and between 2.0 and 2.5 ATA, for three to 6 days a week. The two RCTs by Kessler et al.^37^ and Ma et al.^40^ used the same protocol of 90 minutes at 2.5 ATA twice daily, which enabled them to perform 20 sessions in a short amount of time. These two studies reported the shortest follow‐up periods of four and two weeks, respectively, while the longest follow‐up period was twelve months.^43^ The total numbers of sessions as mentioned in non‐randomized studies ranged from 15 to 110, while both RCTs^37^, ^40^ used a fixed number of 20 sessions. The number of included patients varied greatly between studies. The prospective study by Zamboni et al.^42^ contained 10 patients, while the retrospective study by Margolis et al.^41^ had 6.259 patients.

**Table 1:** Characteristics of the included studies

Author	Year	Study	HBOT size	Control size	Vascularity	HBOT protocol	Number of sessions	Follow‐up	Outcome measures
Akgül	2014	Retrospective	27	‐	Mix	120 mins, 2.4 ATA, 6d/w	mean: 42 (25–110)	12 months	CUH, AR, M
Kessler	2003	RCT	15	13	Non‐ischemic	2x90 mins, 2.5 ATA, 5d/w	20	4 weeks	CUH, AE
Khandelwal	2013	Prospective	20	20	Non‐ischemic	60 mins, 2.5 ATA, 3‐5d/w	30	10 weeks	CUH
Lyon	2008	Retrospective	13	25	Non‐ischemic	NA	NA	8 weeks	US
Ma	2013	RCT	18	18	Non‐ischemic	2x90 mins, 2.5 ATA, 5d/w	20	2 weeks	CUH, M, AE
Margolis	2013	Retrospective	793	5,466	Non‐ischemic	90 mins, 2.0 ATA, 5d/w	median: 29 (15–48)	16 weeks	CUH, AR
Zamboni	1997	Prospective	5	5	Non‐ischemic	120 mins, 2.0 ATA, 5d/w	30	6 months	CUH, AR

Abbreviations: AE, adverse events; AR, amputation rate; ATA, atmosphere absolute; CUH, complete ulcer healing; d/w, days per week; M, mortality; mins, minutes; NA, not available; RCT, randomized controlled trial; US, ulcer size.

### Characteristics of included patients

Table 2 shows the baseline patient characteristics of the included studies. From the study by Akgül et al.,^43^ only the data on patients without PAOD are presented. Margolis et al.^41^ is the only study to report significant differences between groups at baseline, namely for sex, age, ulcer size, and Wagner grade, but for which they did not correct in their analysis. TcpO_2_ was reported by four studies.^37^, ^39^, ^40^, ^42^ Wagner grade, whenever reported, varied among studies. One study included patients with ulcer grades I‐III^40^ and two studies included grades III‐V.

**Table 2:** Baseline patient characteristics

Author		N	Age (mean)	Sex (% male)	Ulcer size (cm^2^)	Duration DM (years)	HbA1c (%)	TcpO_2_ (mmHg)	Wagner grade (% of patients)				
									I	II	III	IV	V
Akgül	HBOT	27	55	70.4	‐	12	‐	‐	‐	‐	92.5	7.5	0
	Control	‐	‐	‐	‐	‐	‐	‐	‐	‐	‐	‐	‐
Kessler	HBOT	14	60.2	71.4	2.31	18.2	9.4	45.2	‐	‐	‐	‐	‐
	Control	13	67.6	69.2	2.82	22.1	8.1	45.6	‐	‐	‐	‐	‐
Khandelwal	HBOT	20	43.8	50	14.91	‐	‐	‐	‐	‐	‐	‐	‐
	Control	20	43.35	55	9.90	‐	‐		‐	‐	‐	‐	‐
Lyon	HBOT	13	69	‐	‐	‐	‐	>30	‐	‐	‐	‐	‐
	Control	25	71	‐	‐	‐	‐	>30	‐	‐	‐	‐	‐
Ma	HBOT	18	59.8	61.1	4.21	24.8	‐	37.06	22.2	22.2	55.6	‐	‐
	Control	18	60.4	66.7	4.35	23.1	‐	35.61	27.8	33.3	38.9	‐	‐
Margolis	HBOT	793	61.6*	64.4*	1.9*	‐	‐	‐	‐	‐	45.7* †	‐	‐
	Control	5,466	63.0	55.7	1.6	‐	‐	‐	‐	‐	18.4†	‐	‐
Zamboni	HBOT	5	63.6	80	6.02	‐	‐	53.4	‐	‐	‐	‐	‐
	Control	5	53.8	80	4.4	‐	‐	60.0	‐	‐	‐	‐	‐

* significant difference.

† Wagner grade ≥ 3.

DM, diabetes mellitus; HbA1c, glycated hemoglobin; HBOT, hyperbaric oxygen therapy; TcpO_2_, transcutaneous oximetry.

### Quality assessment

Because of different HBOT protocols and number of HBOT sessions performed, as well as wound grades and sizes at inclusion, clinical heterogeneity was high.

**Figure 2:**
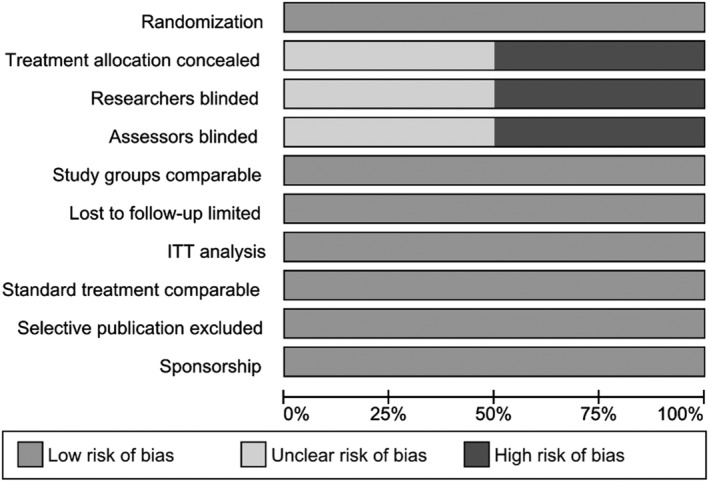
Risk of bias in RCTs.

Figure 2 shows the results of the Cochrane checklist for risk of bias of the randomized studies.^37^, ^40^ The general quality of the RCTs was good, although the authors did not provide adequate information on blinding of the researchers or assessors or stated to have performed an open‐label study.^37^

Table 3 shows the categories for risk of bias for the nonrandomized studies, according to the ROBINS‐I tool. Two studies presented a serious risk of bias due to confounding,^41^, ^42^ while other studies showed a moderate risk of bias in all domains.

**Table 3:** ROBINS‐I checklist for risk of bias

	Confounding	Selection of patients	Classification of interventions	Deviation from intervention	Missing data	Measurement errors	Selective reporting	Overall risk of bias
Akgül	Low	Moderate	Low	Low	Low	Low	Low	Moderate
Khandelwal	Moderate	Low	Low	Low	Low	Moderate	Low	Moderate
Lyon	Moderate	Moderate	Low	Low	Moderate	Low	Moderate	Moderate
Margolis	Serious	Low	Low	Low	Low	Low	Low	Serious
Zamboni	Serious	Moderate	Low	Low	Low	Low	Low	Serious
Overall score	Serious	Moderate	Low	Low	Moderate	Moderate	Moderate	

### Outcome measures

All studies reported on complete ulcer healing, while amputation rate was reported by four studies.^40^, ^41^, ^42^, ^43^ Mortality was reported by two studies.^40^, ^43^ Results of these outcome measures are shown in Table 4. Margolis et al.^41^ did report on complete ulcer healing but reported healing of multiple wounds per patient. A percentage of patients with healed wounds could therefore not be given in Table 4. Lyon et al.^39^ only reported on reduction of ulcer size, which was not a predefined outcome measure because it is less clinically relevant than complete ulcer healing. The two RCTs^37^, ^40^ mention adverse events. Most studies^37^, ^38^, ^40^, ^41^, ^42^, ^43^ reported number of sessions used. None of the included studies reported on QoL, cost of therapy, or amputation‐free survival.

**Table 4:** Outcome measures

Author	HBOT (*N*)	Control (*N*)	Complete ulcer healing, *N* (%)		Amputation rate, *N* (%)				Mortality, *N* (%)	
					Minor		Major			
			HBOT	Control	HBOT	Control	HBOT	Control	HBOT	Control
Akgül	27	‐	16 (59.2)	‐	5 (18.5)	‐	1 (3.7)	‐	5 (18.5)	‐
Kessler	15	13	2 (13.3)	0 (0)	‐	‐	‐	‐	‐	‐
Khandelwal	20	20	12 (60)	8 (40)	‐	‐	‐	‐	‐	‐
Lyon	13	25	0 (0)	0 (0)	‐	‐	‐	‐	‐	‐
Ma	18	18	0 (0)	0 (0)	0 (0)	0 (0)	0 (0)	0 (0)	0 (0)	0 (0)
Margolis	793	5,466	1,210	7,311*	‐	‐	26 (3.28)*	70 (1.28)	‐	‐
Zamboni	5	5	4 (80)*	1 (20)	0 (0)	0 (0)	0 (0)	0 (0)	‐	‐

* Significant difference.

HBOT, hyperbaric oxygen therapy.

### Complete ulcer healing rate

**Figure 3:**
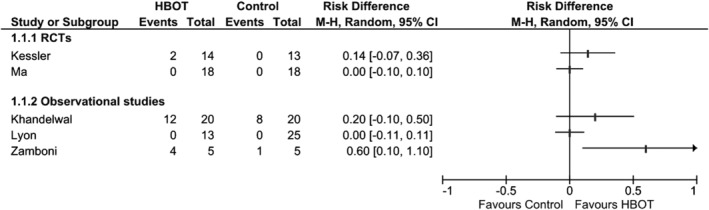
Forest plot of complete ulcer healing results.

Meta‐analysis could not be performed meaningfully for complete wound healing due to clinical heterogeneity. Neither the RCTs^37^, ^40^ nor the nonrandomized studies^38^, ^39^, ^42^ reported significant differences in wound healing. The results are shown in a Forest plot in Figure 3. Akgül et al.^43^ reported that 16 of 27 (59.3%) patients in the HBOT group achieved complete wound healing at the end of the treatment course.

### Major amputation rate

**Figure 4:**
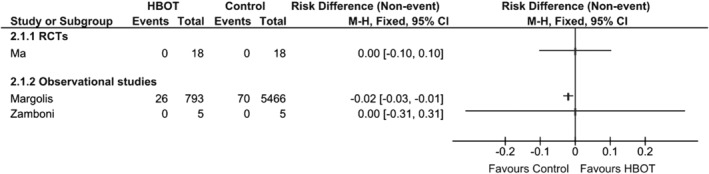
Forest plot of major amputation rates.

Four studies^40^, ^41^, ^42^, ^43^ reported on amputation rates. Meta‐analysis could not be performed meaningfully due to clinical heterogeneity. The only RCT^40^ showed no significant differences, while the large retrospective study by Margolis et al. did show a difference in favor of the control treatment but failed to correct for significant differences between the patient groups at baseline in their analysis. Akgül et al.^43^ reported that 1 out of 27 (3.7%) patients undergoing HBOT needed major amputation in a 24‐month follow‐up. See also the Forest plot in Figure 4.

### Minor amputation rate

Minor amputations were registered by Ma et al.^40^ and Zamboni et al.,^42^ but these did not occur during the follow‐up period of two weeks^40^ and six months,^42^ respectively. In the study by Akgül et al.,^43^ 5 of 27 (18.5%) patients needed minor amputations.

### Mortality rate

Mortality rate was only described in two studies. Ma et al.^40^ reported no deaths during the study period of two weeks and Akgül et al.^43^ reported an 18.5% mortality rate in the HBOT‐treated patients in the 24‐month follow‐up period.

### Adverse events

Adverse events were mentioned in both RCTs. None occurred in the study by Ma et al.^40^ Kessler et al.^37^ reported that 1 of the 14 patients (7.1%) was discharged after developing barotraumatic otitis, which resolved without sequelae.

### Number of sessions performed

The total number of HBOT sessions patients received differ among studies. Most studies^37^, ^38^, ^40^, ^42^ used a fixed number of either 20 or 30 sessions. Akgül et al.^43^ report a range of 25 to 110 sessions and Margolis et al.^41^ report administering 15 to 48 sessions. It is not reported in either study why some patients received less sessions than others. Akgül et al.^43^ mention that most patients were treated as outpatients and Khandelwal et al.^38^ treated patients as inpatients first and later as outpatients (not otherwise defined). Other studies^37^, ^39^, ^40^, ^41^, ^42^ did not mention if study participants were either in‐ or outpatients.

## DISCUSSION

This is the first systematic review that aggregated all available evidence for the specific subgroup of patients with non‐ischemic DFU. From the currently available evidence, it seems that these patients, when treated with HBOT, do not achieve faster wound healing and do not benefit in terms of prevention of major or minor amputations but are at risk of possible HBOT‐related adverse effects. The RCTs that demonstrate this are of good quality. However, the amount of research focusing exclusively on non‐ischemic diabetic ulcers and HBOT is scarce and this result should therefore be viewed with caution. Until additional research has been performed for this specific subgroup, physicians will have to weigh the expected risks and benefits of the therapy and inform patients in this regard, to reach a decision that best fits the patients' preferences.

Earlier systematic reviews, which did not specifically differentiate between the presence or absence of PAOD, arrived at more favorable conclusions concerning wound healing with HBOT. In the Cochrane review on chronic diabetic wounds and HBOT by Kranke et al.,^21^ which included studies with and without PAOD, complete wound healing was achieved more frequently with HBOT at 6 weeks (although no differences were observed anymore after 6 and 12 months). The reviews by Stoekenbroek et al.^26^ and O'Reilly et al.^25^ also included patients with and without PAOD and reported findings similar to those of Kranke et al.^21^ While Stoekenbroek et al.^26^ did not find an increase in complete wound healing, they did report that wound size was reduced in those treated with HBOT. O'Reilly et al.^25^ found some evidence of increased wound healing after HBOT but could not draw definite conclusions from the available evidence. Furthermore, major amputation rate decreased after HBOT in the Cochrane review by Kranke et al.^21^ and in the review by Stoekenbroek et al.^26^ O'Reilly et al^25^ found a decreasing, but not significant trend in both major and minor amputation rates. Possibly, an effect of HBOT may become manifest in DFU patients if PAOD is present, which may account for the different outcomes of the current and earlier reviews. Quantifying the extent of PAOD in patients may help physicians make an informed decision to apply HBOT in wound healing.

In the current review, one large retrospective cohort study^41^ found an increase in major amputation rate for patients treated with HBOT. Since this result is contrary to earlier evidence, it warrants further exploration. Possibly due to the lack of randomization, patients in the HBOT group had a larger wound and more ulcers had a Wagner grade ≥ 3 at inclusion than in the standard wound care group. These patients have an inherently increased risk of major amputation,^28^, ^56^, ^57^ which may have contributed to the observed effect. It also exemplifies the problem of HBOT being used as a “last resort” treatment. In light of this, one of the primary aspects of further research should be concerned with the relationship between HBOT and its timing within the course of treatment.

As indicated in an earlier study by D'Agostino et al.,^58^ 30 sessions of HBOT appear to be the minimally appropriate number of sessions to achieve the desired effect. This was also suggested by the results from another trial.^59^ Therefore, performing less than 30 sessions might decrease the efficacy of HBOT. However, in the included studies,^37^, ^38^, ^39^, ^40^, ^41^, ^42^, ^43^ the number of sessions performed varied. Both RCTs^37^, ^40^ prescribed 20 sessions and report no differences in complete wound healing or either major or minor amputation rate. Margolis et al.^41^ reported an average of 29 sessions, with a range of 15 to 48 sessions. They reported an increase in major amputation rate in the HBOT group. However, they did not report on the number of sessions performed before amputation occurred. In contrast, Khandelwal et al.^38^ used 30 sessions and found a positive, albeit not significant, trend toward complete wound healing, while Zamboni et al.^42^ used 30 sessions as well and reported an increase in complete wound healing after HBOT. These results also suggest that for HBOT to be clinically effective a minimal number of 30 sessions is required.

However, some patients may not be able to complete 30 sessions for various reasons. Protocols with a shorter HBOT‐period might be able to improve patient adherence. However, in the studies by Kessler et al.^37^ and Ma et al.^40^ HBOT was applied twice daily, which led to shorter protocols than other studies used. However, this did not lead to improved wound healing. In fact, complete healing rate was lower than in other studies. Apparently, more than one session of HBOT per day does not lead to better results.

A limitation of this review is the small size and number of RCTs and nonrandomized studies that were available for inclusion. Unfortunately, most authors did not respond to our request for additional data, which also led to the exclusion of two major RCTs.^46^, ^50^ Nevertheless, this review currently represents the best available evidence for this specific subgroup of patients. However, until additional data become available, our results should be interpreted with caution, especially because no meaningful meta‐analyses could be performed due to high clinical heterogeneity.

For future research, we strongly advocate the importance of differentiating between patients with major clinical determinants, such as PAOD. Furthermore, additional patient selection criteria (e.g. TcpO_2_‐values) and (patient‐reported) outcome measures are needed to better phenotype, or identify, patients that might benefit from this treatment modality. The use of a single, standardized HBOT protocol may also facilitate aggregation of study results in future meta‐analyses. Cooperation with healthcare policy makers may help with implementing such a standardized protocol. For the current clinical practice, physicians treating DFUs might also use their patients' characteristics and clinical determinants to help them make an informed decision on the application of HBOT.

In conclusion, the currently available evidence suggests that HBOT does not accelerate wound healing and does not prevent major or minor amputations in patients with DFU without PAOD. Based on this evidence, routine clinical use of this therapy cannot be recommended. However, the available evidence for this specific subgroup of patients is scarce, and physicians should counsel patients on expected risks and benefits. Additional research, focusing especially on patient selection criteria, is needed to better identify patients that might profit from this therapy modality.

## CONFLICT OF INTEREST

The authors declare no conflict of interest in writing this manuscript.

## Appendix A. Complete Search Strategy

MEDLINE (Ovid).

January 1946 – October 1, 2018.

(exp Diabetes Mellitus/ or diabet*.ti,ab,kw.) AND (Hyperbaric Oxygenation/ or ((high* adj3 (pressure or tension*)) and oxygen*).ti,ab,kw. or ((hyperbaric* or barotherap*) and oxygen*).ti,ab,kw. or (HBO or HBOT).ti,ab,kw.) AND (Ulcer/ or exp Leg Ulcer/ or “Wounds and Injuries”/ or diabetic foot/ or (ulcer* or wound* or diabetic foot).ti,ab,kw.) Embase (Ovid).

January 1947 – October 1, 2018.

(exp diabetes mellitus/ or diabet*.ti,ab,kw.) AND (hyperbaric oxygen therapy/ or ((high* adj3 (pressure or tension*)) and oxygen*).ti,ab,kw. or ((hyperbaric* or barotherap*) and oxygen*).ti,ab,kw. or (HBO or HBOT).ti,ab,kw.) AND (ulcer/ or exp skin ulcer/ or ulcer healing/ or leg ulcer/ or exp wound/ or diabetic foot/ or (ulcer* or wound* or diabetic foot).ti,ab,kw.) Cochrane Library.

Up to October 2018.

(diabet*:ti,ab,kw OR [Diabetes Mellitus]) AND ([Hyperbaric Oxygenation] OR (high* near/3 (pressure or tension*)) AND oxygen*:ti,ab,kw OR (hyperaric* OR barotherap*) AND oxygen*:ti,ab,kw OR HBO OR HBOT:ti,ab,kw) AND (ulcer* OR wound* OR diabetic foot:ti,ab,kw)
